# Reality of treatment for severely injured patients: are there age-specific differences?

**DOI:** 10.1186/s12873-024-00935-w

**Published:** 2024-01-24

**Authors:** Teresa Maek, Ulrike Fochtmann, Pascal Jungbluth, Bastian Pass, Rolf Lefering, Carsten Schoeneberg, Sven Lendemans, Bjoern Hussmann

**Affiliations:** 1 Department of Orthopedics and Trauma Surgery, Alfried Krupp Hospital, Alfried-Krupp-Straße 21, 45131 Essen, Germany; 2 Department of Orthopedics and Trauma Surgery, University Hospital Duesseldorf, Moorenstraße 5, 40225 Duesseldorf, Germany; 3 Institute for Research in Operative Medicine (IFOM), Herdecke University, Ostmerheimer Straße 200, 51109 WittenCologne, Germany; 4 University of Duisburg-Essen, Hufelandstraße 55, 45122 Essen, Germany

**Keywords:** Trauma registry, Severely injured patients, Age factors, Matched triplet analysis, Outcome

## Abstract

**Background:**

Major trauma and its consequences are one of the leading causes of death worldwide across all age groups. Few studies have conducted comparative age-specific investigations. It is well known that children respond differently to major trauma than elderly patients due to physiological differences. The aim of this study was to analyze the actual reality of treatment and outcomes by using a matched triplet analysis of severely injured patients of different age groups.

**Methods:**

Data from the TraumaRegister DGU® were analyzed. A total of 56,115 patients met the following inclusion criteria: individuals with Maximum Abbreviated Injury Scale > 2 and < 6, primary admission, from German-speaking countries, and treated from 2011–2020. Furthermore, three age groups were defined (child: 3–15 years; adult: 20–50 years; and elderly: 70–90 years). The matched triplets were defined based on the following criteria: 1. exact injury severity of the body regions according to the Abbreviated Injury Scale (head, thorax, abdomen, extremities [including pelvis], and spine) and 2. level of the receiving hospital.

**Results:**

A total of 2,590 matched triplets could be defined. Traffic accidents were the main cause of severe injury in younger patients (child: 59.2%; adult: 57.9%). In contrast, low falls (from < 3 m) were the most frequent cause of accidents in the elderly group (47.2%). Elderly patients were least likely to be resuscitated at the scene. Both children and elderly patients received fewer therapeutic interventions on average than adults. More elderly patients died during the clinical course, and their outcome was worse overall, whereas the children had the lowest mortality rate.

**Conclusions:**

For the first time, a large patient population was used to demonstrate that both elderly patients and children may have received less invasive treatment compared with adults who were injured with exactly the same severity (with the outcomes of these two groups being opposite to each other). Future studies and recommendations should urgently consider the different age groups.

## Background

Serious accidents and their consequences are one of the leading causes of death worldwide at any age, and even the leading cause between the ages of 5 and 29 [1]. Current guidelines for the care of severely injured patients have been based on scientific studies of younger, male patients in particular [2, 3]. However, there has been a steady increase in older trauma patients for decades (increase in patients > 70 years between 2015 and 2020 from 23 to 29%) [4].

In all age groups, major trauma has a significant impact on morbidity and mortality. It is therefore surprising that, on the one hand, there are few age-specific guidelines and, on the other hand, little age-comparative literature addressing cause, diagnosis, and therapy in the age groups. In some cases, there are major differences, which are based on different physiology in children and elderly [5].

Differences between younger and elderly are also found in the causes of accidents: in younger patients, traffic accidents (high impact trauma), in elderly falls from low height (low impact trauma) are the main cause of accidents [6–9].

In contrast to elderly, children up to 16 years of age still represent a rarity after severe accident, but especially at this age the consequences of a severe accident are the main cause of death [1, 10]. In the current TraumaRegister® annual report, only 3.5% severely injured children have been documented, thus complicating a scientific discussion [4]. Comparable to elderly, however, is that recommendations and guidelines based on large study collectives are rare in children. The same applies to specific scientific analyses on outcome-relevant parameters in the different age groups, possibly different diagnostics (e.g., avoidance of radiation exposure in children), and possibly therapy adapted to an advance directive in especially elderly [11, 12]. For example, studies have shown that whole-body computed tomography (CT) can positively influence the outcome of severely injured patients, but especially in very young children, a targeted examination of, for example, the head in CT is more likely to be performed, whereas a whole-body CT is not performed without negatively influencing the outcome [13–15].

Due to inconsistencies in the current literature and, in particular, insufficient data taking into account patient age, this study investigated whether there are age-specific differences in the care of severely injured patients. An analysis of the causes of accidents, initial diagnosis and therapy was followed by an outcome analysis between age groups.

## Methods

The TraumaRegister DGU® of the German Trauma Society (Deutsche Gesellschaft für Unfallchirurgie, DGU) was founded in 1993. The aim of this multi-center database was to provide pseudonymized and standardized documentation of severely injured patients.

The data is collected prospectively in the following four consecutive time phases from the site of the accident until discharge from the hospital: A) Prehospital phase; B) Emergency room and initial surgery; C) Intensive care unit (ICU); and D) Discharge. The documentation includes detailed information on demographics, injury pattern, co-morbidities, pre- and in-hospital management, progression in the intensive care unit, and relevant laboratory findings including data on transfusion and the outcome of each individual patient. The inclusion criterion is hospital admission via the emergency room with subsequent ICU or hospital arrival with vital signs and death before admission to the ICU. The infrastructure for documentation, data management, and data analysis is provided by the Academy for Trauma Surgery (AUC—Akademie der Unfallchirurgie GmbH), a company that is affiliated with the German Trauma Society. The scientific leadership is provided by the Committee on Emergency Medicine, Intensive Care and Trauma Management (Sektion NIS) of the German Trauma Society. The participating hospitals submit their data pseudonymized into a central database via a web-based application. The scientific data analysis is approved according to a peer review procedure established by Sektion NIS. The participating hospitals (90%) are primarily located in Germany; however, an increasing number of hospitals from other countries (such as Austria, Belgium, China, Finland, Luxembourg, Slovenia, Switzerland, The Netherlands, and the United Arab Emirates) also contribute data. Currently, the data for over 28,000 patients from nearly 700 hospitals have been entered into the database annually. Participation in the TraumaRegister DGU® is voluntary. For hospitals associated with the TraumaNetzwerk DGU®, however, the entry of at least one basic data set is obligatory for reasons of quality assurance (this part of the methodology was described earlier in [16, 17]).

The present study is consistent with the publication guidelines of the TraumaRegister DGU® (TR-DGU) and is registered under the TR-DGU project ID 2019–057. Because of pseudonymous retrospective data analysis using TraumaRegister DGU®, waiver of informed consent was obtained from the ethics committee of the Medical Association of North Rhine, Tersteegenstraße. 9, Duesseldorf, Germany (internal number: 165/2022).

To ensure reliable comparability of the data, only patients from participating hospitals in Germany, Austria, and Switzerland were included in the study.

Sepsis (as a life-threatening [multi]organ failure condition) was calculated by using the Sequential Organ Failure Assessment (SOFA) score. The following parameters were used for the calculation: PaO_2_/FiO_2_, Glasgow Coma Scale (GCS), mean arterial blood pressure (MAP) or use of vasopressors, bilirubin, platelets, and creatinine [18]. A score ≥ 3 on the SOFA indicated organ failure [19]. If two independently affected organ systems exhibited pathology, this scenario was included in the analysis as multiorgan failure (MOF).

When considering the collected parameters, the following parameters were only available from 2016: the administration of tranexamic acid, pelvic binder, coagulopathy existing before the accident, death due to termination of therapy based on a living will within 7 days after admission to the hospital, and the administration of calcium in the emergency room.

**Fig. 1:**
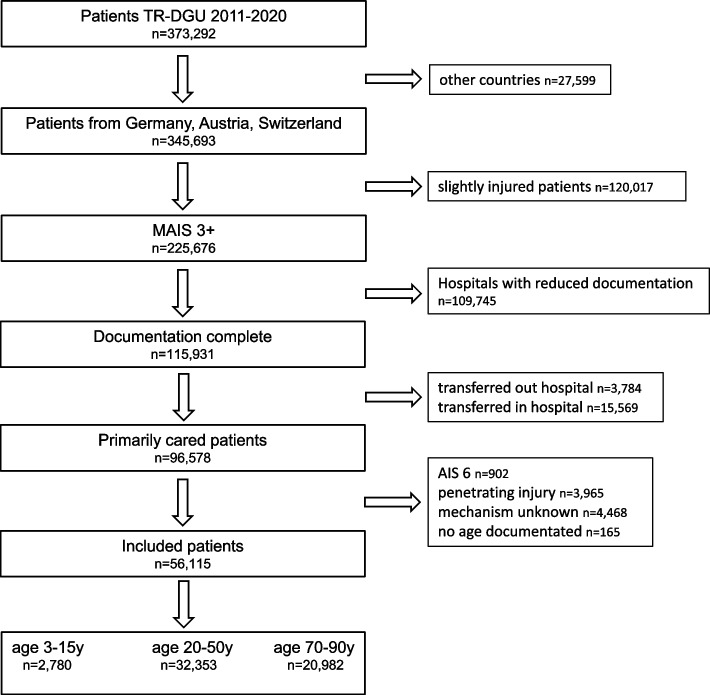
Graphic representation of the test procedure

The following severely injured patients of different age groups that were documented in the TR-DGU from 2011 to 2020 who met the following inclusion criteria were analyzed (Fig. 1):

1. patients from Germany, Austria, and Switzerland
2. primary admitted patients (who were not transferred out within 48 h after admission)
3. Maximum Abbreviated Injury Scale (MAIS) > 2 and < 6
4. available age
5. blunt trauma
6. standard documentation (including several interventions)

As shown in Fig. 1, according to their ages, the severely injured patients were further divided into three groups: child (3–15 years), adult (20–50 years), and elderly (70–90 years) groups. This classification of groups was chosen to ensure a clear delineation of the age groups. Therefore, the interpretation of the data should be more clearly age-specific. Thus, 56,115 patients met the inclusion criteria.

To evaluate the influence of age on severely injured patients with an identical injury pattern, matched triplets of patients from different age groups were performed based on the following criteria:

- The exact injury severity of the different body regions (head, thorax, abdomen, extremities [including the pelvis], and spine) by using the Abbreviated Injury Scale (AIS) = 0–1, 2, 3, 4, or 5.
- The level of receiving hospital, according to certification by DGU: (1) supra-regional trauma center, (2) regional trauma center, and (3) local trauma center.

The matched triplet criteria were selected in order to allow for reliable comparability between the groups in terms of injury severity. In this context, no injury was present with an AIS of 0 in the corresponding body region. Minor injuries (AIS = 1) were disregarded and merged with the none category. This step was performed to clarify the focused analysis of severe injuries. The level of care of the receiving hospital (levels 1–3) was also included as a matching criterion; this was done to ensure that different levels of care of the hospitals (for example, due to the presence of other specialties) did not have an influence on the analysis, thus affecting the results.

### Statistical analysis

Analysis was performed by using the Statistical Package for the Social Sciences (SPSS; version 24, IBM Inc., Chicago, IL, USA). Incidences are expressed as the numbers of cases with percentages. Continuous variables are presented as the mean with standard deviation (SD) or as the median with interquartile range, as appropriate. Formal statistical testing of the differences in the groups was not performed because the very large number of cases would make even small and clinically irrelevant differences statistically significant. With this number of cases, differences of approximately 2% (depending on the prevalence) would become "significantly" different in the pairwise comparisons; in addition, for metric variables, a difference of approximately one-twentieth of the standard deviation would indicate significance. In the present study, only complete data from the TR were included in the analysis; missing data were not replaced, but only the existing and thus valid data were analyzed and reported. All data sets showed at least a completion rate between 90–95%.

## Results

### General descriptive data

A total of 2,590 patients per group were available for the matched triplet analysis and could be further investigated. In contrast to the adults with 77.8% male patients, the gender distribution was more balanced in the children and elderly (Table 1). Injury severity scores (ISS) were comparable across all three groups (Table 1). A reduced Glasgow Coma Scale (GCS) score of ≤ 8 points was least common in the elderly group at 21.2%. In contrast, the proportion of pre-diseased patients according to the American Society of Anesthesiologists (ASA) scores of 3 and 4 points was most common in the elderly patients (child: 0.8%; elderly: 41.5%).

**Table 1:** Descriptive age-specific data from severely injured patients

Group	Children (*n* = 2,590)	Adults (*n* = 2,590)	Elderly (*n* = 2,590)
Male (%)	63.6	77.8	55.3
Age in years (MV, SD)	10.1 ± 3.9	35.2 ± 9.6	78.5 ± 5.5
ISS (MV, SD)	18.5 ± 9.1	19.0 ± 9.2	18.7 ± 9.1
Prehospital GCS ≤ 8 (%)	24.5	26.1	21.2
ASA 3–4 (%)	0.8	4.6	41.5

Values are the mean, standard deviation (SD) or % of the group; ISS, Injury Severity Score; GCS, Glasgow Coma Scale

ASA, American Society of Anesthesiologists

The results of the matched-triplet criteria (injury severity and level of first-care hospital) established by the methodology are shown in Table 2.

**Table 2:** Prevalence of matching criteria in all three subgroups (*n* = 7,770; *n* = 2,590 per group)

**AIS head: (%)**	
0	36.6
2	9.7
3	22.0
4	20.8
5	10.8
AIS thorax: (%)	
0	64.7
2	9.0
3	19.4
4	5.4
5	1.4
AIS abdomen: (%)	
0	84.3
2	7.5
3	4.8
4	2.7
5	0.6
AIS extremities including pelvis: (%)	
0	48.6
2	19.2
3	29.4
4	2.5
5	0.3
AIS spine: (%)	
0	86.6
2	8.0
3	3.9
4	0.3
5	1.2
Hospital level (%)	
1	88.3
2	10.8
3	0.8

Values are the % of the group; Abbreviated Injury Scale, AIS

With the exception of the elderly, traffic accidents were the main cause of serious injury (child: 59.2%; adult: 57.9%; elderly: 37.1%). In addition to bicycle accidents, pedestrian accidents were more frequent among children. In the elderly group, falls from a low height < 3 m (low falls) were most frequent (Table 3).

**Table 3:** Causes of accidents and accident history in group-specific comparison

Group	Children (*n* = 2,590)	Adults (*n* = 2,590)	Elderly (*n* = 2,590)
**Traffic accident (%)**	**59.2**	**57.9**	**37.1**
**Type of accident (%)**			
**Motor vehicle**	10.6	24.4	11.6
**Motorcycle**	6.2	16.6	3.2
**Bicycle**	16.4	9.6	10.4
**Pedestrian**	23.1	6.2	11.0
**Fall:**			
**High ≥ 3 m**	17.4	16.8	10.8
**Low < 3 m**	13.1	12.9	47.2
**Other**	13.2	13.4	5.8

Values are % of the group

### Prehospital diagnostics and treatment

At initial diagnosis, the results were comparable across all groups. Only the children tended to have weaker blood pressure, increased heart rate and more patients in hemorrhagic shock (child: RRsys < 90 mmHg: 12.5%). The GCS was again almost equally distributed across all groups (Table 4).

**Table 4:** Prehospital diagnostics and initial therapy in group comparison

Group	Children (*n* = 2,590)	Adults (*n* = 2,590)	Elderly (*n* = 2,590)
BP at accident site (mmHg)	116 ± 25	127 ± 29	141 ± 38
BP ≤ 90 mmHg (%)	12.5	9.4	10.5
Heart rate at accident site (beats/min)	100 ± 25	90 ± 23	85 ± 20
GCS at the accident site	11.7 ± 4.4	11.6 ± 4.5	11.9 ± 4.1
Prehospital interventions			
Number of interventions in total	1.9 ± 1.1	2.1 ± 1.1	1.8 ± 1.1
Volume replacement (%)	88.0	92.6	88.6
Average amount of volume replacement (ml)	588 ± 452	866 ± 631	667 ± 494
Intubation (%)	34.4	37.1	29.0
Sedation (%)	71.5	72.3	58.6
Chest tube (%)	1.5	2.5	1.7
Pelvic binder (%) [recorded from 2016]	10.6	15.3	9.3
Administration of catecholamines (%)	6.9	7.8	8.6
Administration of tranexamic acid (%) [recorded from 2016]	9.3	14.2	7.8
CPR (%)	4.2	3.2	2.7
On-scene time (min)	28 ± 16	29 ± 17	27 ± 15

Values are the mean, standard deviation (SD) or % of the group; BP blood pressure; GCS Glasgow Coma Scale;

CPR cardiopulmonary resuscitation

Similar results were obtained in the analysis of prehospital measures (Table 4). Here, there was an average of just under 2 prehospital measures per group. Thus, about 90% of the patients in all three groups received prehospital volume, although the children's group received the least prehospital volume (child: 587.6 ml, adult: 866.1 ml, elderly: 666.5 ml). With the exception of resuscitation at the scene (where children were most prevalent) and administration of catecholamines (where elderly were most prevalent), all other prehospital measures were given most frequently in adults for initial therapy (Table 4). Time on scene was again similarly distributed across all groups.

### Initial diagnostics and treatment in the hospital

Compared to the other two groups, 39.9% more children had been transported to the hospital by helicopter (Table 5).

**Table 5:** Initial diagnosis and therapy in the hospital in the group comparison

Group	Children (*n* = 2,590)	Adults (*n* = 2,590)	Elderly (*n* = 2,590)
Prehospital time from accident to hospital admission (min)	66 ± 29	67 ± 31	70 ± 42
Helicopter transport (%)	39.9	32.2	23.4
Hospital admission at weekend (%)	45.3	47.4	38.5
Hospital admission at night (%)	38.6	42.9	31.6
BP in hospital (mmHg; MV, SD)	118 ± 23	128 ± 27	138 ± 35
Volume replacement in hospital (ml; MV, SD)	800 ± 1012	1181 ± 1389	924 ± 1059
Intubation in the emergency room (%)	12.1	11.4	9.5
CPR emergency room (%)	2.1	1.8	2.3
Catecholamines in the emergency room (%)	11.3	15.6	17.6
Chest tube in the emergency room (%)	3.9	7.1	5.6
Coagulopathy (Quick’s value ≤ 60%, or INR ≥ 1.4, or PTT ≥ 40 s) (%)	13.9	9.6	25.5
BE (MV, SD)	-2.0 ± 4.1	-2.1 ± 4.4	-1.7 ± 4.8
Hb at admission (g/dl)	12.0 ± 1.9	13.3 ± 2.2	12.2 ± 2.3
Quick (%)	79.6 ± 16.3	87.9 ± 18.2	78.1 ± 26.9
INR	1.2 ± 0.5	1.1 ± 0.3	1.4 ± 0.9
Preexisting coagulation disorder (%) [recorded from 2016]	0.3	1.0	50.2
Blood transfusion in the emergency room (%)	7.3	8.7	10.0
Mass transfusion (≥ 10 units of pRBC; %)	0.5	1.2	0.9
FFP administration emergency room (%)	3.1	5.2	4.1

Values are the mean, standard deviation (SD) or % of the group; BP,blood pressure; CPR, cardiopulmonary resuscitation;

INR, International Normalized Ratio; PTT, Prothrombin time; BE, Base excess; Hb, haemoglobin;. pRBC, packed red blood cells; FFP, fresh-frozen plasma

Regarding blood pressure values in the emergency room and emergency measures, such as intubation, the results were comparable to those of the prehospital phase. Adults also received most diagnostic and therapeutic measures in the emergency room. Also with the exception of resuscitation and administration of catecholamines.

**Fig. 2:**
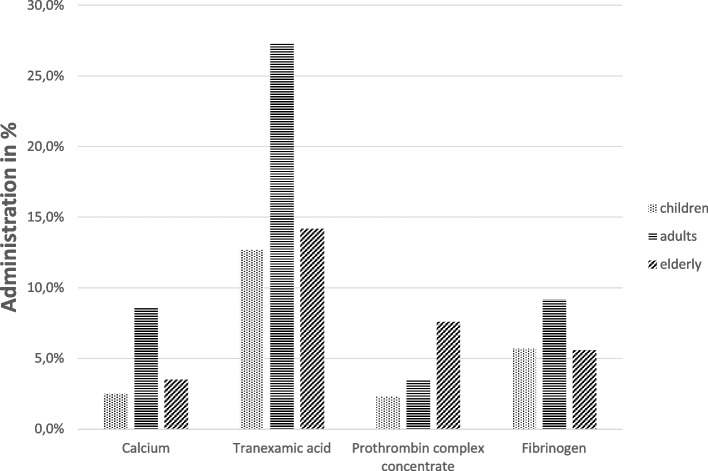
Administration of coagulants in the emergency room in group comparison. (Tranexamic acid and calcium indication recorded from 2016)

As shown in Table 5, the elderly had coagulopathy at hospital arrival to a significantly greater extent (25.5%). Similarly, the proportion of coagulopathies already existing before the accident was highest in this group, at 50.2%. Correspondingly, the elderly were more treated with prothrombin complex concentrate (PPSB) (Fig. 2). The other laboratory parameters from Table 5 (hemoglobin, International Normalized Ratio, prothrombin time, Quick) indicating hemorrhage or coagulopathy were similarly distributed across the groups analyzed. The same was true for the administration of erythrocyte concentrates (EC) and fresh frozen plasma (FFP). Only the administration of tranexamic acid occurred most frequently in the adults.

**Fig. 3:**
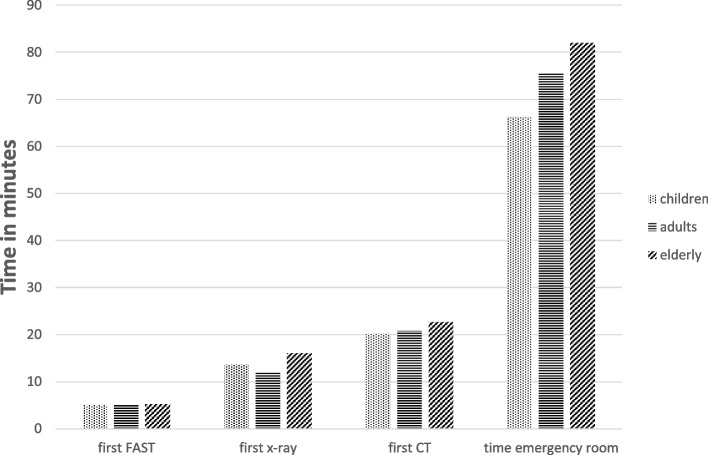
Time to radiological measures in the emergency room and total emergency room time

With regard to radiological diagnostics, it was found that, with the exception of sonography (FAST), CT in particular was performed the least in the children, both selectively for the head and as a whole-body CT (Fig. 3). The times of a radiological examination were again similarly distributed across the groups. The total time in the emergency room was longest in the elderly.

### Clinical course and outcome

The transfer of the patient from the emergency room (e.g., ICU) and emergency surgery or surgery can be seen in Table 6. Overall, the clinical course showed that the group of elderly patients had the worst course in all parameters studied, such as sepsis, organ failure (OF), MOF, and thromboembolic events, when compared between groups (Table 6).

**Table 6:** Clinical course and outcome in group comparison

Group	Children (*n* = 2,590)	Adults (*n* = 2,590)	Elderly (*n* = 2,590)
Transfer from ER to (%):			
OP	39.8	41.2	27.6
ICU/IMC	49.4	50.8	60.1
other hospital	0.3	0.5	0.2
others	6.2	3.4	8.2
Emergency surgery (%)	29.5	25.8	16.3
Surgery (%)	63.3	70.1	63.4
Sepsis (%)	2.1	4.3	6.4
Organ failure (%)	22.4	33.5	44.2
Multiple organ failure (%)	12.1	18.8	25.6
Thrombembolic events (%)	0.8	1.5	4.6
Expected hospital mortality rate based on RISC II (%)	6.2	7.0	20.1
Died in hospital (%)	5.6	7.1	21.5
Died in the emergency room (%)	1.7	1.4	1.3
Died witih 24 h after admission in hospital (%)	3.7	4.0	9.7
Died due to termination of therapy based on living will within 7 days after admission to hospital (%) [recorded from 2016]	0.2	0.2	1.5
Outcome (%):			
Vegetative state	1.1	1.5	2.2
Severe disability	5.9	8.2	11.9
Moderate disability	17.7	24.9	31.4
Good recovery	75.4	65.4	54.4

Values are the mean, standard deviation (SD) or % of the group; ICU, intensive care unit; IMC, Intermediate Care;;

RISC, Revised Injury Severity Classification

**Fig. 4:**
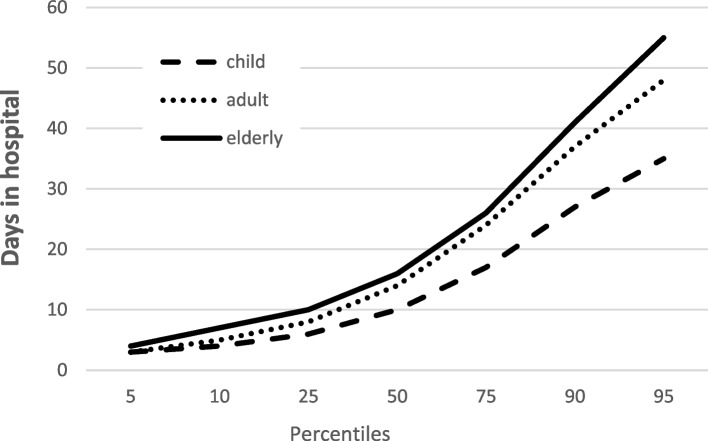
Distribution of total hospital length of stay in severely injured children, adults, and elderly individuals

A comparable result was found for the percentiles of length of stay. Children were represented with the fewest days of ventilation (95th percentile: children 12 days, adults 20 days, elderly 22 days), days in the intensive care unit (95th percentile: children 22 days, adults 27 days, elderly 32 days), as well as total hospitalization time (Fig. 4). When analyzing the Glasgow Outcome Scale (GOS), it was the elderly who had a worse outcome (Table 6).

Similarly, the overall mortality rate was highest in the elderly group with 21.5%, although in this group, with 1.5%, therapy was discontinued more frequently due to the patient's will. However, early mortality (death in the emergency room) tended to be highest in children, at 1.7% (Table 6).

## Discussion

In the results of this study, the reality of the treatment of severely injured patients of different ages in a large collective sample was demonstrated for the first time with the aid of a matched triplet analysis. Specifically, age-specific differences in the mechanism of the accident, the initial diagnosis and therapy, or the outcome could be demonstrated.

It is well known that male patients are particularly likely to be severely injured after an accident [4, 20]. However, it is striking that the gender difference is smaller in both children and elderly patients and may even be reversed in the elderly. This was also demonstrated by Gioffrè-Florio et al. in their study on elderly patients [21]. A possible explanation why more female patients are found as severely injured patients with increasing age could be the higher average life expectancy in women [22]. Otherwise, an increased general tendency to take risks, an profession with more accident potential, or the practice of high-risk sports, in younger male adults in particular, is a possible explanation for the higher proportion in this group. Accordingly, Rugg et al. saw increased male patients in snowboarding accidents, as an example of a high-risk sport [23]. When considering the causes of accidents, it is notable that in the group of children up to 15 years of age, falls from both low (< 3 m) and greater heights (> 3 m) are initially present, and with increasing age and mobility, traffic accidents as cyclists or pedestrians are predominant [24, 25].

When considering the initial diagnostic values at the scene, such as blood pressure and pulse rate, it should be noted that the children had decreased blood pressure and increased pulse rate. The proportion of children with signs of hemorrhagic shock (RRsys < 90 mmHg) was also greatest across all groups. However, it must always be kept in mind that smaller children, in particular, have lower blood pressure and increased heart rate due to their physiological normal values, and thus a pathological value was not necessarily present [5]. In contrast, in the elderly patients, preexisting cardiovascular medications (e.g., heart frequency decreasing medications) may be the cause of a lower heart rate at the scene of the accident, thus masking an initial hemorrhagic shock and delaying potentially life-saving therapy [26]. In this study, the proportion of patients with signs of hemorrhagic shock (RRsys < 90 mmHg) tended to be even higher in the elderly patients than in the comparison group of adults. However, when discussing these results, it is important to keep in mind that especially in the group of elderly patients, undiagnosed cardiovascular diseases may have an influence on the initially measured blood pressure or heart rate, which may affect pre-existing hypertension or hypotension and may further complicate the initial diagnosis [27]. Moreover, it should be mentioned that in a retrospective analysis based on pseudonymized data, no conclusion can be drawn about the individual patient. Thus, only possible correlations (but no absolute causalities) can be discussed.

A remarkable finding of this study is that, despite exactly the same injury severity existing in the different body regions and despite almost identical overall injury severity (according to the ISS), elderly received fewer measures (both prehospital and in the emergency room) compared with adults, with the exception of the administration of catecholamines, although the total number of prehospital measures was almost equally distributed across all of the groups with approximately 2 measures. However, when considered individually, it is remarkable that only prehospital resuscitation and prehospital administration of sedating drugs were performed the least amount in the elderly group. Although it has been well studied that outcome worsens with age, an altered immunologic response to severe trauma in the elderly patient may be the cause [17, 28, 29]. Emergency procedures that may reduce mortality in younger patients, such as thoracotomy, do not seem to improve outcomes in patients over 57 years of age, so that emergency surgery was performed less in the elderly in our study [30]. Nevertheless, van der Sluis et al. clarified that they could not find any arguments against equal treatment of elderly compared with younger patients in their retrospective analysis [31]. However, current guidelines such as the S3 polytrauma guideline do not specifically address different age groups [3]. Thus, as a rule, only recommendations exist that have been studied in an adult collective and are ultimately transferred to other age groups. As mentioned above, it remains speculative in a retrospective analysis why elderly received fewer measures, especially prehospital, for the same injury severity. It is also possible that injuries in elderly patients following accidents with rather low kinetics (falls < 3 m) are initially underestimated by the emergency team, although these may lead to higher injury severity in elderly patients [32]. A similar conclusion of this "under triage" was reached by Ricard-Hibon et al. in their study. They also found evidence in the literature that elderly are even more likely to benefit from more aggressive therapy at the scene of the accident and concluded that therapy limitation should only occur after arrival at the hospital [33].

Interestingly, this occurrence of "under triage" is also observed in children after a severe injury. Thus, in the present study, prehospital measures were also less performed in children compared with adults. Brooke Lerner et al. in their recent study even saw an increase in "under triage" over the years in the preshospital setting after severe trauma in children [34]. However, in contrast to elderly "under triage," children are intubated more and transported to a trauma center by helicopter more often with shorter rescue times. This is all well described already [35]. Here, transport to a trauma center specific for children may be the reason. It is also important to note that children were resuscitated more often prehospital and in the emergency room despite exactly the same injury severity. Why this is especially the case in children must remain speculative on the basis of this retrospective study. A possible explanation could be that the acting emergency team in children, even with injuries with simultaneous cardiovascular arrest that are prognostically unlikely to survive, nevertheless transports these patients to a trauma center. This assumption is supported by the data presented here. For example, more patients in the children's group tended to die early in the emergency room. This could be an indication of more incompatible injuries. It should be noted that only patients who reach a hospital are included in the TR-DGU. This fact would support the hypothesis that more children were hospitalized despite nonsurvivable injury compared with elderly and thus could be documented in the TR-DGU. Another possible explanation is that children are postulated to have a fundamentally better outcome after resuscitation for trauma in comparison [36, 37]. Teeter et al. even described children as a positive predictive factor for better outcome after resuscitation and trauma [38]. The data presented here support this association. Thus, the incidence of sepsis, organ failure, and multiple organ failure was significantly less in children than in the two adult collectives. When children survived the first 24 h after trauma, subsequent mortality was lowest and outcome according to the GOS was best. Therefore, it is not surprising that ICU length of stay or total hospital length of stay was shortest in the children and longest in the elderly patients. However, with regard to mortality in the elderly, it must be kept in mind that a larger proportion of therapies were discontinued due to the patient's wishes compared to the younger patients.

In summary, it remains remarkable that the adults received more in almost all diagnostic and therapeutic measures in the group comparison, both in the prehospital phase and in the in-hospital phase with exactly the same injury severity. And even though their measured diagnostic scores at the scene tended to be even better. Thus, fewer patients were in hemorrhagic shock (RRsys < 90 mmHg) among adults at the scene. It also remains unclear why, with the exception of the administration of PPSB, fewer coagulants such as tranexamic acid or fibrinogen were administered in the elderly patients. Although, as might be expected, elderly had more preexisting coagulation disorders anyway. Although PPSB has been well studied in the literature as an antagonist for oral anticoagulants in the elderly patient, e.g., after a severe TBI, and thus may be more likely to be used in practice [39]. It remains unexplained why elderly patients received less tranexamic acid, for example. Especially since it has been known since the CRASH-2 trial that tranexamic acid may improve mortality, especially within the first hour after trauma [40].

On the other hand, the fact that children received fewer radiological diagnostic tools such as CT is reasonable. In particular, the saving of a radio exposure in children plays a crucial role here. Huber-Wagner et al. were able to show that performing a whole-body CT scan can improve outcome, but these data referred to adult patients [13]. In contrast, Abe et al. concluded that a selective CT scan, for example of the head, saves radiation and does not increase mortality in children [41]. It remains unclear why elderly also received fewer CT examinations. It is possible that more frequent treatment discontinuations occurred in this group because a non-survivable injury was already detected on CT of the head. However, this must remain unclear due to a lack of current literature on this point and the retrospective nature of this study.

Finally, it must be considered that a comparison of injury severities according to the AIS of body regions between the different age groups, does not necessarily trigger an exact same therapeutic pathway. It is conceivable, for example, that a pulmonary contusion in an adult may require more therapeutic effort to treat than in comparably injured children. For example, Evans et al. demonstrated that the AIS severity score does not represent the variability of functional impairment at discharge [42]. Nevertheless, the AIS represents an internationally recognized score that is applied and used scientifically across all ages. Unlike the GCS, for example, an AIS adapted only for children is not yet available. Nevertheless, this should be considered in principle and taken into account in future guidelines.

### Limitations

1. In a strictly retrospective analysis based on pseudonymized data, it is not possible to clarify the individual decision of the acting hospital team. Additionally, access to the patient record for further analysis was not possible due to pseudonymization.
2. Only possible links with conclusions can be described in the examined data (not absolute causalities).
3. Only patients who could be transferred to a hospital or emergency room are recorded in the TR-DGU. Patients who died at the scene of the accident are not documented; therefore, they cannot be evaluated.
4. All of the patients were treated on site by a physician. However, it remains unclear in this analysis which specialization the physician possessed (e.g., anesthesiologist, surgeon, etc.).
5. No data on the health and medical history of the patients were available in the TR-DGU.

## Conclusion

For the first time, it could be shown on the basis of a large patient collective that despite exactly the same injury severity, a matched triplet group analysis of children, adults and elderly patients reveals considerable differences with regard to the initial diagnosis and care after a severe trauma. Thus, adults seem to be treated more according to current guidelines, which is expressed by the majority of measures taken at the scene and in the emergency room. For children and elderly patients, on the other hand, there is evidence of "under triage," although this is different for children and elderly. For example, children receive more resuscitations at the scene. Children also have a better outcome after major trauma. Future guidelines need to take into account different age groups and their changing physiology.

Therefore, further prospective randomized studies are urgently needed to further investigate the question investigated here with a higher level of evidence.

## Data Availability

All data generated or analyzed during this study are included in this published article.

## Abbreviations

AIS: Abbreviated Injury Scale
ASA: American Society of Anesthesiologists
AUC: Academy for Trauma Surgery
BP: Blood pressure
CPR: Cardiopulmonary resuscitation
CT: Computed tomography
DGU: German Association for Trauma Surgery
FAST: Focused Assessment with Sonography for Trauma
FFP: Fresh frozen plasma
FiO_2_: Fraction of inspired oxygen
GCS: Glasgow Coma Scale
HEMS: Helicopter emergency medical services
ICU: Intensive Care Unit
IMC: Intermediate Care
INR: International Normalized Ratio
ISS: Injury Severity Score
MAIS: Maximum Abbreviated Injury Scale
MAP: Mean arterial blood pressure
MOF: Multiple organ failure
MV: Mean Value
NIS: Committee on Emergency Medicine, Intensive Care and Trauma Management
OF: Organ failure
PaO_2_: Partial pressure of arterial oxygen.
PCC: Prothrombin complex concentrate
pRBC: Packed red blood cells
PTT: Prothrombin time
RISC II: Revised Injury Severity Classification II
SD: Standard Deviation
SOFA: Sequential Organ Failure Assessment
SPSS®: Statistical Package for the Social Sciences
TR-DGU: TraumaRegister DGU®
TBI: Traumatic Brain Injury
WHO: World Health Organization

## References

[1] WHO. Many of the leading causes of death for adolescents and young adults are injuries. Injuries are categorized by whether the injury was intentional (for example, homicide) or unintentional (for example, road traffic crashes). Geneva: WHO. https://platform.who.int/mortality/themes/theme-details/MDB/injuries. Accessed 19 Jan 2023.

[2] Demetriades D, Kimbrell B, Salim A, Velmahos G, Rhee P, Preston C (2005). Trauma deaths in a mature urban trauma system: is "trimodal" distribution a valid concept?. J Am Coll Surg.

[3] AWMF Online. Guidelines of polytrauma (S3) of the German society of trauma surgery. http://www.awmf.org/leitlinien/aktuelle-leitlinien/ll-liste/deutsche-gesellschaft-fuer-unfallchirurgie-ev.html. Accessed 19 Jan 2023.

[4] Allgemeine jahresberichte des traumaregister DGU® seit dem Jahr 2000. Jahresbericht 2021. http://www.traumaregister-dgu.de/de/service/downloads.html. Accessed 19 Jan 2023.

[5] Silverman BK, Fleisher GR, Ludwig S, Henretig FM (2006). Textbook of pediatric emergency medicine. Textbook of pediatric emergency medicine.

[6] Meisler R, Thomsen AB, Theilade P, Abildstrøm H, Borge P, Treschow M (2011). Age-related differences in mechanism, cause, and location of trauma deaths. Minerva Anestesiol.

[7] Lesko K, Deasy C (2020). Low falls causing major injury: a retrospective study. Ir J Med Sci.

[8] Jawa RS, Singer AJ, Rutigliano DN, McCormack JE, Huang EC, Shapiro MJ (2017). Spinal fractures in older adult patients admitted after low-level falls: 10-year incidence and outcomes. J Am Geriatr Soc.

[9] Lee H, Kim SH, Lee SC, Kim S, Cho GC, Kim MJ (2018). Severe injuries from low-height falls in the elderly population. J Korean Med Sci.

[10] Paneitz DC, Ahmad S (2018). Pediatric trauma update. Mo Med.

[11] Marco CA, Michael S, Bleyer J, Post A (2015). Do-not-resuscitate orders among trauma patients. Am J Emerg Med.

[12] Salottolo K, Offner PJ, Orlando A, Slone DS, Mains CW, Carrick M (2015). The epidemiology of do-not-resuscitate orders in patients with trauma: a community level one trauma center observational experience. Scand J Trauma Resusc Emerg Med.

[13] Huber-Wagner S, Lefering R, Qvick LM, Körner M, Kay MV, Pfeifer KJ (2009). Effect of whole-body CT during trauma resuscitation on survival: a retrospective, multicentre study. Lancet.

[14] Meltzer JA, Stone ME, Reddy SH, Silver EJ (2018). Association of whole-body computed tomography with mortality risk in children with blunt trauma. JAMA Pediatr.

[15] Garcia CM, Cunningham SJ (2018). Role of clinical suspicion in pediatric blunt trauma patients with severe mechanisms of injury. Am J Emerg Med.

[16] Hussmann B, Schoeneberg C, Jungbluth P, Heuer M, Lefering R, Maek T (2019). Enhanced prehospital volume therapy does not lead to improved outcomes in severely injured patients with severe traumatic brain injury. BMC Emerg Med.

[17] Hussmann B, Heuer M, Lefering R, Touma A, Schoeneberg C, Keitel J (2015). Prehospital volume therapy as an independent risk factor after trauma. Biomed Res Int.

[18] Singer M, Deutschman CS, Seymour CW, Shankar-Hari M, Annane D, Bauer M (2016). The third international consensus definitions for sepsis and septic shock (Sepsis-3). JAMA.

[19] Vincent JL, Moreno R, Takala J, Willatts S, De Mendonça A, Bruining H (1996). The SOFA (Sepsis-related organ failure assessment) score to describe organ dysfunction/failure. On behalf of the working group on sepsis-related problems of the European society of intensive care medicine. Intensive Care Med.

[20] Ilie G, Trenholm M, Boak A, Mann RE, Adlaf EM, Asbridge M (2020). Adolescent traumatic brain injuries: onset, mechanism and links with current academic performance and physical injuries. PLoS ONE.

[21] Gioffrè-Florio M, Murabito LM, Visalli C, Pergolizzi FP, Famà F (2018). Trauma in elderly patients: a study of prevalence, comorbidities and gender differences. G Chir.

[22] Rau R, Schmertmann CP (2020). District-level life expectancy in Germany. Dtsch Arztebl Int.

[23] Rugg CD, Malzacher T, Ausserer J, Rederlechner A, Paal P, Ströhle M (2021). Gender differences in snowboarding accidents in Austria: a 2005–2018 registry analysis. BMJ Open.

[24] Laurer H, Wutzler S, Wyen H, Westhoff J, Lehnert M, Lefering R (2009). Quality of prehospital and early clinical care of pediatric trauma patients of school age compared to an adult cohort. A matched-pair analysis of 624 patients from the DGU trauma registry. Unfallchirurg.

[25] McAdams RJ, Swidarski K, Clark RM, Roberts KJ, Yang J, McKenzie LB (2018). Bicycle-related injuries among children treated in US emergency departments, 2006–2015. Accid Anal Prev.

[26] Evans DC, Khoo KM, Radulescu A, Cook CH, Gerlach AT, Papadimos TJ (2014). Pre-injury beta blocker use does not affect the hyperdynamic response in older trauma patients. J Emerg Trauma Shock.

[27] Whiteman C, Davidov DM, Sikora R, Paulson D, Schaefer G (2016). Major trauma and the elder west virginian: a six year review at a level I trauma center. W V Med J.

[28] Soles GL, Tornetta P (2011). Multiple trauma in the elderly: new management perspectives. J Orthop Trauma.

[29] Mörs K, Wagner N, Sturm R, Störmann P, Vollrath JT, Marzi I (2021). Enhanced pro-inflammatory response and higher mortality rates in geriatric trauma patients. Eur J Trauma Emerg Surg.

[30] Gil LA, Anstadt MJ, Kothari AN, Javorski MJ, Gonzalez RP, Luchette FA (2018). The national trauma data bank story for emergency department thoracotomy: how old is too old?. Surgery.

[31] Van Der Sluis CK, Klasen HJ, Eisma WH, ten Duis HJ (1996). Major trauma in young and old: what is the difference?. J Trauma.

[32] Sammy I, Lecky F, Sutton A, Leaviss J, O'Cathain A (2016). Factors affecting mortality in older trauma patients-A systematic review and meta-analysis. Injury.

[33] Ricard-Hibon A, Duchateau FX, Vivien B (2012). Out-of-hospital management of elderly patients for trauma injury. Ann Fr Anesth Reanim.

[34] Lerner EB, Cushman JT, Drendel AL, Badawy M, Shah MN, Guse CE (2017). Effect of the 2011 revisions to the field triage guidelines on under- and over-triage rates for pediatric trauma patients. Prehosp Emerg Care.

[35] Ashburn NP, Hendley NW, Angi RM, Starnes AB, Nelson RD, McGinnis HD (2020). Prehospital trauma scene and transport times for pediatric and adult patients. West J Emerg Med.

[36] Zwingmann J, Mehlhorn AT, Hammer T, Bayer J, Südkamp NP, Strohm PC (2012). Survival and neurologic outcome after traumatic out-of-hospital cardiopulmonary arrest in a pediatric and adult population: a systematic review. Crit Care.

[37] Perron AD, Sing RF, Branas CC, Huynh T (2001). Predicting survival in pediatric trauma patients receiving cardiopulmonary resuscitation in the prehospital setting. Prehosp Emerg Care.

[38] Teeter W, Haase D (2020). Updates in traumatic cardiac arrest. Emerg Med Clin North Am.

[39] Joseph B, Hadjizacharia P, Aziz H, Kulvatunyou N, Tang A, Pandit V (2013). Prothrombin complex concentrate: an effective therapy in reversing the coagulopathy of traumatic brain injury. J Trauma Acute Care Surg.

[40] Roberts I, Shakur H, Afolabi A, Brohi K, Coats T, Dewan Y (2011). The importance of early treatment with tranexamic acid in bleeding trauma patients: an exploratory analysis of the CRASH-2 randomised controlled trial. Lancet.

[41] Abe T, Aoki M, Deshpande G, Sugiyama T, Iwagami M, Uchida M (2019). Is whole-body CT associated with reduced in-hospital mortality in children with trauma? A nationwide study. Pediatr Crit Care Med.

[42] Evans LL, Jensen AR, Meert KL, VanBuren JM, Richards R, Alvey JS, Carcillo JA, McQuillen PS, Mourani PM, Nance ML, Holubkov R, Pollack MM, Burd RS (2022). All body region injuries are not equal: Differences in pediatric discharge functional status based on Abbreviated Injury Scale (AIS) body regions and severity scores. J Pediatr Surg.

